# Comparative cost-effectiveness of cabozantinib as second-line therapy for patients with advanced hepatocellular carcinoma in Germany and the United States

**DOI:** 10.1186/s12876-020-01241-y

**Published:** 2020-04-21

**Authors:** Maximilian Sieg, Michael Hartmann, Utz Settmacher, Habibollah Arefian

**Affiliations:** 1 Faculty of Medicine, Friedrich Schiller University Jena, Jena, Germany; 2 Center for Sepsis Control and Care (CSCC), Jena University Hospital, Jena, Germany; 3 Hospital Pharmacy, Jena University Hospital, Jena, Germany; 4 Department of General, Visceral and Vascular Surgery, Jena University Hospital, Jena, Germany

**Keywords:** Hepatocellular carcinoma, Cost-effectiveness, Cabozantinib, Second-line therapy

## Abstract

**Background:**

Cabozantinib was approved by the European Medicines Agency and the Federal Drug Administration as an option for sorafenib-resistant advanced hepatocellular carcinoma, increasing overall survival and progression-free survival compared with placebo. We evaluated the cost-effectiveness of cabozantinib in the second-line setting for patients with an advanced hepatocellular carcinoma from the German statutory health insurance perspective compared with an US scenario using US prices.

**Methods:**

A Markov model was developed to compare the costs and effectiveness of cabozantinib with best supportive care in the second-line treatment of advanced hepatocellular carcinoma over a lifetime horizon. Health outcomes were measured in discounted life years and discounted quality-adjusted life years. Survival probabilities were estimated using parametric survival distributions based on CELESTIAL trial data. Utilities were derived from the literature. Costs contained drugs, monitoring and adverse events measured in US Dollars. Model robustness was addressed in univariable, scenario and probabilistic sensitivity analyses.

**Results:**

Cabozantinib generated a gain of 0.18 life years (0.15 quality-adjusted life years) compared with best supportive care. The total mean cost per patient was $56,621 for cabozantinib and $2064 for best supportive care in the German model resulting in incremental cost-effectiveness ratios for cabozantinib of $306,778/life year and $375,470/quality-adjusted life year. Using US prices generated costs of $177,496 for cabozantinib and $4630 for best supportive care and incremental cost-effectiveness ratios of $972,049/life year and $1,189,706/quality-adjusted life year.

**Conclusions:**

Our analysis established that assuming a willingness-to-pay threshold of $163,371/life year (quality-adjusted life year) for the German model and $188,559/life year (quality-adjusted life year) for the US model, cabozantinib is not cost-effective compared with best supportive care. Sensitivity analyses showed that cabozantinib was not cost-effective in almost all our scenarios.

## Background

Hepatocellular carcinoma (HCC) has a constantly increasing incidence in Germany and the United States, as developed countries, with approximately 6628 (8.2/100,000 inhabitants, Germany) and 24,223 (7.7/100,000 inhabitants, United States) new diagnoses in 2012 [1, 2]. Former hepatitis B and C infections, alcoholic cirrhosis and especially the increasing risk of nonalcoholic steatohepatitis are the main drivers of its development [1]. The relative 5-year survival rate remains low at 15%, and curative options such as resection and transplantation are suitable only for locally limited HCC [3, 4]. Therefore, advances in systemic therapy for advanced HCC are highly necessary.

Since the breakthrough of sorafenib enhanced the treatment of HCC, patients have suffered a lack of second-line options after progression. Brivanib and everolimus failed to improve overall survival (OS) versus placebo in phase III trials [5, 6]. In 2017, regorafenib showed a significant OS prolongation from 7.8 months to 10.6 months versus placebo, leading to approvals by the European Medicines Agency (EMA) and US Food and Drug Administration and its implementation in the European and US market as the first second-line option for systematic HCC therapy [7].

The Federal Joint Committee (GBA) is the German statutory authority that assesses the degree of benefit of newly approved therapies and decides on their reimbursement by the statutory health insurances (SHI). They acknowledged the drug as a nonquantifiable added benefit in the previously approved indication of third-line treatment for advanced colorectal cancer. As a consequence, the pharmaceutical producer Bayer withdrew regorafenib from German markets because they had identified no opportunity to achieve a sufficient reimbursement amount [8]. Therefore, oncologists could not provide the expensive regorafenib therapy for HCC in Germany, because its costs were no longer reimbursed.

In June 2019, the GBA voted to follow the recommendations of the German Institute for Quality and Efficiency in Health Care (IQWiG) acknowledging a minor added benefit of cabozantinib in the second-line therapy of HCC after progression with sorafenib [9]. Cabozantinib is a tyrosine kinase inhibitor (TKI) that targets tyrosine kinases, such as vascular endothelial growth factor receptor (VEGFR) and hepatocyte growth factor receptor (MET) and it has already been approved as a first-line treatment for advanced medullary thyroid carcinomas and advanced renal cell carcinomas (RCC). This appraisal is based on the findings of Abou-Alfa et al. regarding the CELESTIAL trial results [10, 11]. Patients treated with cabozantinib showed an OS of 10.2 months compared with 8.0 months with placebo. Progression-free survival (PFS) was prolonged from 1.9 to 5.2 months [10].

National healthcare systems are facing rising costs to provide new effective therapies. Our aim is to determine the cost-effectiveness of cabozantinib therapy compared with best supportive care (BSC) in patients whose HCC was inadequately treated by sorafenib within the context of the German and the US healthcare system. We considered only BSC as a comparator in the model, as other second-line systemic HCC drugs were not available or reimbursable in Germany or didn’t match the target population. Our BSC definition includes health items of monitoring, consulting and adequate treatment of adverse events. The cabozantinib group received equal BSC and cabozantinib. The primary outcome measure is the incremental cost-effectiveness ratio (ICER) quantified in $ per life year (LY) and $ per quality-adjusted LY (QALY). We assumed cost-effectiveness thresholds estimated as multiples of the gross domestic product (GDP) per capita deduced from the 3 times GDP per capita rule of the WHO [12]. The impact of uncertainties on the ICER was investigated in sensitivity analyses.

## Methods

### General

The selection of data sources and methods follow the German health economic evaluation recommendations of the IQWiG [13]. Our Markov model was implemented in TreeAge Healthcare Pro 2019 software (TreeAge Pro 2019, R1.1; *TreeAge Software, Williamstown, MA*). The data were obtained from published material of the CELESTIAL trial and the submitted GBA dossier of IPSEN Pharma and completed by a literature review on cabozantinib, TKIs and HCC [14]. We considered these sources as adequate clinical effectiveness data, because the CELESTIAL trial met its primary endpoint, was sufficiently reported and 48% of the trial subjects are from Europe and 24% from the United States or Canada [10].

### Target population, setting and perspective

Our target population in the model was based on the CELESTIAL trial subjects: adult patients with HCC who showed progression under prior sorafenib therapy. The methods of the CELESTIAL trial were described in the published study protocol [15]. In summary, 707 patients were randomized into the cabozantinib group or placebo group and were treated with 60 mg cabozantinib per day or the placebo. The inclusion of patients with Child-Pugh A liver function and the exclusion of patients with an uncontrolled clinically significant illness allowed only relatively healthy patients to be included the trial population [15]. We used published data with reference to other TKIs treating HCC if it was necessary to fill data gaps because of identical drug classes and comparable side effects.

88% of people in Germany were insured by SHIs in 2015 and 12% by private health insurances [16]. The US population is covered by multiple overlapping insurance forms: 55.4% of patients are insured employment-based, 10.8% use direct-purchase and the public plans Medicare and Medicaid cover 17.8 and 17.9% [17]. Furthermore, 8.5% were insured [17]. We chose the perspective of German SHIs for costs induced by cabozantinib therapy and BSC. As a consequence of the diverse US insurance forms and the resulting difficultness to measure exemplary costs, we quantified the chosen health item of the German SHI perspective with adequate US equivalents described in Cost calculation.

### Model structure

**Fig. 1:**
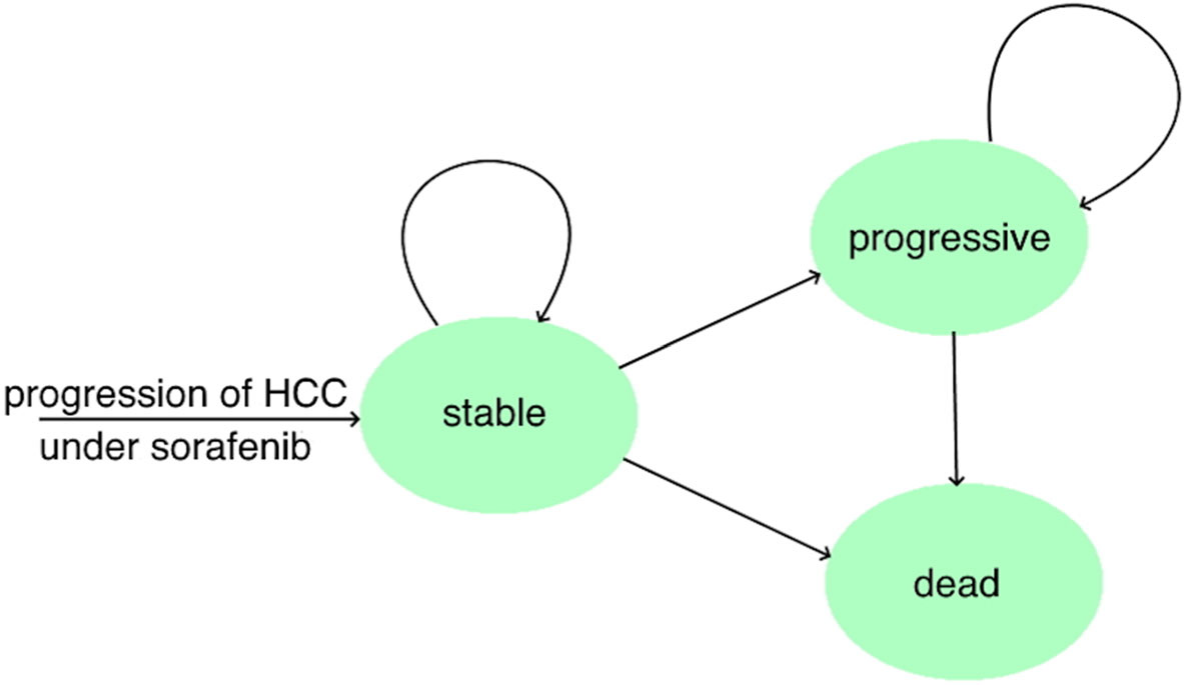
State transition diagram. HCC = hepatocellular carcinoma

A Markov model was constructed to estimate the costs and utility gains of the target population illustrated with Fig. 1. It consists of three health states representing the natural process of HCC: stable, progressive and dead. All patients started in stable and either stayed at that stage or transitioned to progressive or dead. The transition to progressive represents the CELESTIAL trial progression definition [15]. Once in the progressive stage, patients were able only to either remain in that stage or to die.

### Time horizon, cycle length and discounting

A time lifetime horizon (84 months) was selected to simulate a lifetime horizon providing a practically complete cost and utility estimation of a therapy in a palliative setting. A 30-day cycle length was adopted. The discounting of costs and utilities was performed with a rate of 3%, as recommended by the IQWiG [13].

### Transition probabilities

Transition probabilities were deduced from published Kaplan-Meier (KM) curves of the CELESTIAL trial. Parametric distributions were fitted to the KM data for PFS and OS via minimizing the sum of squared residuals to extrapolate from the trial duration to the 84-month horizon. We followed the recommendations of Ishak et al. fitting five parametric distributions to the KM data: exponential, Weibull, Gompertz, log-logistic and lognormal [18]. The best-fitting distribution for all curves was Weibull selected via the sum of squared residuals, Akaike information criterion, Bayesian information criterion and the long-time hazard of the distribution, matching the characteristics of a palliative setting. Goodness of fit values are presented in Table 1 and the detailed transition probabilities and distribution parameters are described in Additional file 1.

**Table 1:** Estimated distribution goodness of fit values of progression-free survival and overall survival

Distribution	Weibull^a^	Exponential^b^	Gompertz^a^	Loglogistic^c^	Lognormal^d^
PFS cabozantinib					
SSR	0,012	0,026	0,016	0,021	0,015
AIC	−188	−170	− 180	− 173	− 181
BIC	− 185	− 168	−178	−171	− 179
OS cabozantinib					
SSR	0,010	0,032	0,016	0,019	0,014
AIC	− 337	− 292	− 317	−312	− 322
BIC	− 334	− 290	− 314	− 308	− 319
PFS BSC					
SSR	0,070	0,080	0,080	0,035	0,039
AIC	− 143	− 142	− 140	− 160	−158
BIC	− 140	−140	− 137	− 158	− 155
OS BSC					
SSR	0,121	0,161	0,154	0,021	0,014
AIC	− 208	− 199	−199	− 273	− 287
BIC	−205	−197	− 196	− 270	− 283

*PFS* progression-free survival, *OS* overall survival, *BSC* best supportive care, *SSR* sum of squared residuals, *AIC* Akaike information criterion, *BIC* Bayesian information criterion. ^a^ Monotonically increasing. ^b^ Constant hazard. ^c^ Increasing followed by a gradually decreasing hazard. ^d^ Hazard increases to a maximum and then decreases to 0 as time tends to infinity

### Utilities

Evaluating quality of life (QoL) represents an essential step in determining the effectiveness of novel therapies with high rates of adverse events (AEs). Abou-Alfa et al. published the differences in mean total QALYs during cabozantinib treatment with a significant increase of 0.092 for the entire follow-up using the EQ-5D-5 L QoL questionnaire without reporting total QALYs. The study was limited by low questionnaire return numbers (82–100%) [19]. In our base case, we used 0.76 for stable and 0.68 for progressive. These estimations refer to the findings of Thomsen et al. about QALYs in sorafenib-treated RCC and were used in many cost-effectiveness analyses, including the submission about sorafenib for HCC to the British National Institute for Health and Care Excellence (NICE) [20–22]. The findings of Bruix et al. examining the QALYs of the RESORCE population under regorafenib therapy support these values (0.76 under regorafenib and 0.77 under placebo) [23]. As described under *Target Population, Setting and Perspective,* we found it appropriate to use these values. QALY reductions by AEs were not included into the base case QALYs, because the high AE rates would lead to lower QALYs in the cabozantinib group compared with BSC disagreeing with the QoL findings of Abou-Alfa et al. described above.

### Cost calculation

#### General

We considered direct medical costs, including drug, monitoring and AE costs using $ for easier comparison, converted by purchasing power parities of 2019 with 0.741 €/$ and 0.689 £/$ as the exchange rates [24]. The frequency and forms of supporting health items were primarily deduced from German clinical practice guidelines and completed by the study protocol recommendations [15].

The German SHI perspective requires health items to be matched with the German diagnosis related groups (DRG) system for hospitalizations and the German Uniform Value Scale catalog for outpatient procedures [25]. DRG values were estimated using the DRG-Research Group Webgrouper. Drug prices and reimbursement amounts were deduced from the pharmacy database Lauer-Taxe of 15th April 2019.

In contrast to Germany, US prescription drug prices have no standardized maximum prices and are affected by multiple rebates and reimbursement programs. We determined the model costs using the US drug price portal GoodRX.com via extracting the average cash prices in April 2019. We estimated physician outpatient fees, other services and hospitalizations using the 2019 physician fee schedule, clinical laboratory fee schedule and Medicare-Severity DRG classifications and software (HCPCS-DRG V1.0 Software) of Centers for Medicare and Medicaid Services and the methods of Tumeh et al. [26].

#### Costs of cabozantinib medication

Producers of patented drugs and the SHIs negotiate discounts for every newly approved drug in Germany regarding the acknowledged added benefit by the GBA and the costs of appropriate alternative therapies through a process structured by the Pharmaceuticals Market Reorganization Act (AMNOG). If a drug provides multiple indications, such as cabozantinib treating thyroid carcinoma, RCC and HCC, a single discount must represent all indications. The list price of 30 portions of cabozantinib of all dosages amounts to $8461, and the current reimbursement amount is $6841. Dose modifications were not considered in the model because 40 mg and 20 mg pills produce similar costs in Germany. Therefore, we incorporated the current AMNOG amount of cabozantinib in our German model. The GoodRX.com price for 30 portions of cabozantinib of 60 mg was $21,581 in April 2019.

According to the CELESTIAL trial protocol, cabozantinib was applied until radiographic progression or discontinuation induced by high-grade AEs [15]. The median time to cabozantinib discontinuation (3.8 months) and the rate of discontinuation owing to AEs related to the cabozantinib trial regime (16%) were the only available published data on the time to discontinuation [10]. Regarding the lack of explicit data on the discontinuation rate over time and the minority of toxicity-related discontinuations, we considered it appropriate to include cabozantinib medication costs in all stable months.

#### Costs of disease monitoring

The German HCC guideline and the CELESTIAL study protocol recommend dynamic contrast-enhanced magnetic resonance imaging or computed tomography scan every 2 months as follow-up imaging [15, 27]. The mean costs of both imaging methods were used to match these conditions. The chosen laboratory panel was constructed following the German HCC guidelines on sorafenib for HCC [27].

#### Costs of treating adverse events

Our model included treatment-related AEs of every grade reported in ≥5% of patients in either treatment arm in the CELESTIAL trial. We performed a chi-squared test to exclude AEs that demonstrated no significant (*p* < 0.05) difference between the two intervention groups to decide whether it was caused by cabozantinib (see Additional file 1). Furthermore, clinically related AEs such as nausea and vomiting were summarized. Required health items included drugs, outpatient services and hospitalizations and were determined along the grade definitions of the Common Terminology Criteria for Adverse Events (CTCAE), Version 4.3. We considered no differences between both comparator groups regarding types, frequencies and costs of the chosen health items. AEs that did not require reimbursable therapy were excluded. The included AE costs were the sum of every grade costs multiplied with its grade incidence.

Adverse events requiring permanent therapy (e. g. hypertension grade 1 requiring antihypertensive drugs) were treated with health items using daily standard dosages with 30 doses per cycle and were matched with an adequate drug compound. The amounted costs were included into all stable cycles until progression. If an adverse events needs a defined treatment with a temporal limit and no need of repetition (e.g. urinary tract infections grade 1 requiring antibiotics for a standardized period), the matching drug compound and its costs were only included into cycle 1 of the state stable. Adverse event grades requiring hospitalizations (e.g. hypertension grade 4, a hypertensive emergency) were matched to an adequate DRG. As we assumed only one hospitalization case per adverse event per patient, we included the costs only in cycle 1 of state stable. All estimated costs per item of both countries are listed in Table 2 below.

**Table 2:** Monthly Cost Summary

	Costs in $ per month^a^			
Perspective	Germany		United States	
Item	Cabozantinib	BSC	Cabozantinib	BSC
Cabozantinib drug	6841	0	21,581	0
Consultation	37	37	110 (75)	110 (75)
Laboratory	27 (14)	27 (14)	110 (55)	110 (55)
Imaging	93	93	162	162
AE total	682 (139)	213 (52)	1673 (645)	557 (166)
Diarrhea	271 (13)	48 (4)	444 (24)	80 (8)
Hand-foot-syndrome	42 (29)	3 (2)	385 (315)	25 (20)
Fatigue	15	9	93	56
Nausea and vomiting	93 (45)	64 (26)	168 (77)	116 (44)
Hypertension	17 (12)	2	78 (70)	11
Abdominal pain	24 (0)	41 (0)	122 (0)	187 (0)
Stomatitis	4	1	7	1
Rash	16	8	52	27
Thrombocytopenia	32 (0)	0	78 (0)	0
Dyspepsia	2 (0)	1 (0)	8 (0)	2 (0)
Hypokalemia	88 (1)	24 (< 1)	121 (3)	33 (1)
Pain in extremity	2 (0)	1 (0)	1 (0)	< 1 (0)
Hypothyroidism	13 (< 1)	< 1	21 (3)	< 1
Hypomagnesemia	33 (0)	0	45 (0)	0
Urinary tract infection	26 (0)	11 (0)	45 (0)	19 (0)

The listed AE costs are already incidence-weighted. *AE* adverse event, *BSC* best supportive care. ^a^ Deviating costs of second and following months in brackets

### Cost-effectiveness thresholds

Because Willingness-to-pay described through ICERs stays difficult to measure and both national healthcare systems have no cost-effectiveness thresholds, we estimated cost-effectiveness thresholds as multiples of the GDP per capita deduced from the 3 times GDP per capita rule of the WHO [12]. The primary cost-effectiveness threshold was the 3 times GDP per capita per gained LY or QALY and was completed by factor 6 and 9 for a broader overview. Table 3 shows the most current GDPs and the deduced thresholds [28].

**Table 3:** Estimated cost-effectiveness thresholds

	Germany	United States
GDP per capita in $ in 2018	54,457	62,853
Factor	Cost-effectiveness thresholds in $/LY (QALY)	
3	163,371	188,559
6	326,742	377,118
9	490,113	565,677

*GDP* gross domestic product, *LY* life year, *QALY* quality-adjusted life year

### Sensitivity analyses

#### Cost analyses

The impact of the difference between the median PFS (5.2 months) and median time to discontinuation of cabozantinib (3.8 months) was investigated in two ways. First, cabozantinib therapy was applied for only four standardized cycles (months) even without progression. The second approach was subtracting a monthly cabozantinib price if a patient suffered progression to simulate the case of progression 1 month after discontinuation.

#### Utility analyses

The high grade of uncertainty regarding QoL under cabozantinib therapy as described in *Utilities* was investigated through multiple values from findings on QoL regarding HCC and TKI. Approaches and utilities are presented in Table 5. Despite the findings of Abou-Alfa et al. indicating a QoL gain through cabozantinib, we examined the case of AE-induced disutilities in contrast to our base case. Grade 3/4 incidences of the most important treatment-related AEs were multiplied with disutility values deduced primarily from Kobayashi et al. and complementary to that of Lloyd et al. to calculate reduced QALY values during stable disease [30, 31]. A last method was to use a monthly decreasing utility in a progressive state simulating constantly decreasing QoL after progression.

#### Univariant and probabilistic sensitivity analyses

A univariant sensitivity analysis varied major model inputs at defined intervals to determine their impact. Our probabilistic sensitivity analyses were conducted to explore the likelihood of cabozantinib reaching cost-effectiveness by varying major model inputs simultaneously in 10,000 iterations. We used a gamma distribution for costs following the recommendations of the IQWiG to use a distribution with positive skewness and no upper limit [13]. The IQWiG recommended discounting rate borders of 0 and 5% for sensitivity analyses [13]. These rates were implemented via a beta distribution providing the defined borders and a mean of 3%. A normal distribution offered a simple option to include determined confident intervals of utilities into the probabilistic sensitivity analysis. Detailed distribution data are presented in the Additional file 1.

## Results

### Base case results

Therapy with cabozantinib compared with BSC resulted in a gain of 9.4 weeks or 2.2 months of life (0.18 LYs). Adjusted for QoL, using cabozantinib led to a gain of 0.15 QALYs. Cabozantinib therapy resulted in $56,621 and BSC in $2064 in Germany and $177,496 versus $4630 in the US model. The utility gain and costs are itemized in Table 4. The ICERs for cabozantinib versus BSC were $306,778/LY and $375,470/QALY gained in Germany and the US model resulted in ICERs of $972,049/LY and $1,189,706/QALY.

**Table 4:** Base case utility and cost breakdown

	Cabozantinib	BSC	
Utilities			
	Utility gain	Utility gain	Incremental utility
Stable LY	0.646	0.341	0.305
Progressive LY	0.505	0.632	−0.127
Total	1.151	0.973	0.178
Stable QALY	0.491	0.259	0.232
Progressive QALY	0.343	0.429	−0.086
Total	0.834	0.698	0.146
	Costs in $ (%^a^)	Costs in $ (%^a^)	Incremental costs (%^a^)
Costs Germany			
Cabozantinib	53,018 (93.6)	0 (0.0)	53,018 (97.2)
Adverse events	1607 (2.8)	375 (18.2)	1232 (2.3)
Consultation	513 (0.9)	434 (21.0)	69 (0.1)
Laboratory	202 (0.4)	173 (8.4)	29 (< 0.1)
Imaging	1281 (2.3)	1083 (52.5)	198 (0.4)
Total	56,621 (100.0)	2064 (100.0)	54,556 (100.0)
Costs United States			
Cabozantinib	167,288 (94.6)	0 (0.0)	167,288 (96.8)
Adverse events	6030 (3.8)	1075 (56.2)	4955 (2.9)
Consultation	1075 (0.6)	914 (43.8)	161 (0.1)
Laboratory	868 (0.3)	751 (0.0)	117 (0.1)
Imaging	2236 (0.7)	1890 (0.0)	346 (0.2)
Total	177,496 (100.0)	4630 (100.0)	172,866 (100.0)

*BSC* best supportive care, *LY* life years, *QALY* quality-adjusted life years. Total values may be affected by rounding errors. ^a^ % of total

### Sensitivity analyses

**Fig. 2:**
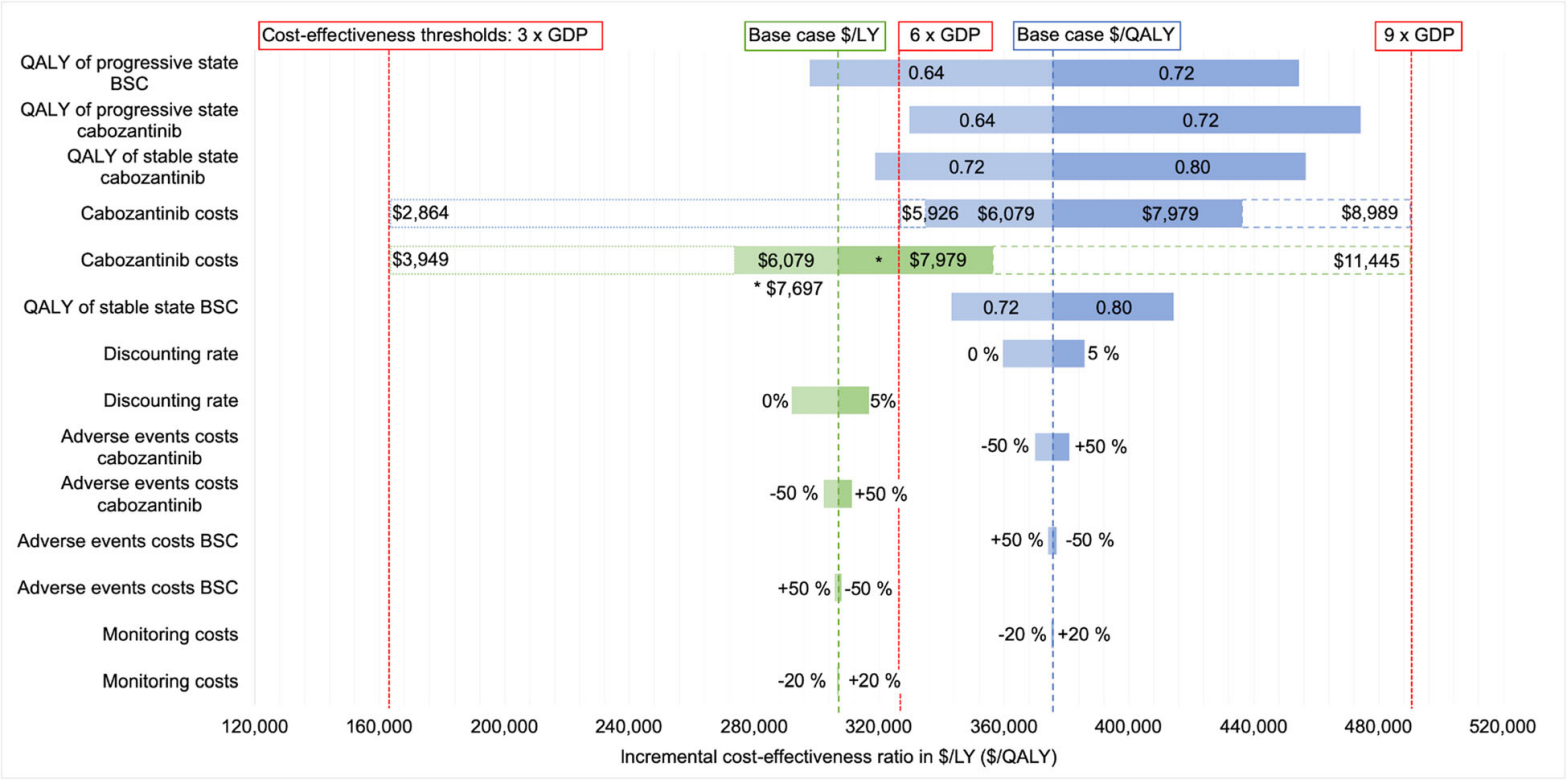
Tornado diagram of univariant sensitivity analyses German model. GDP = gross domestic product per capita; LY = life years; QALY = quality-adjusted life years; BSC = best supportive care

**Fig. 3:**
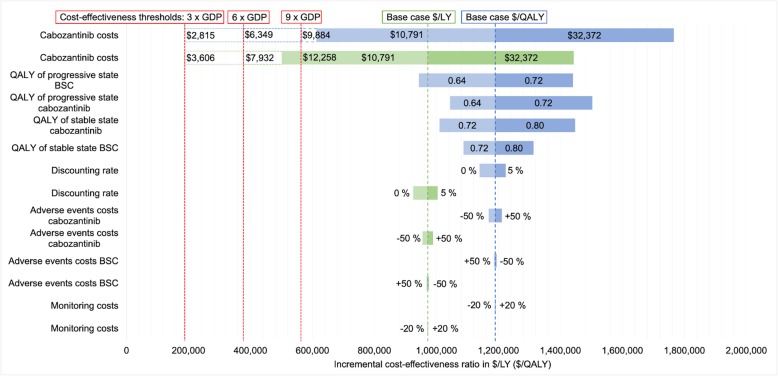
Tornado diagram of univariant sensitivity analyses US model. GDP = gross domestic product per capita; LY = life years; QALY = quality-adjusted life years; BSC = best supportive care

The results of our univariant and cost-threshold sensitivity analyses are illustrated with tornado diagrams in Figs. 2 and 3. Even with broad variation in the ranges of each parameter, the ICERs remained over the 3 times GDP per capita/LY (QALY) threshold. Monitoring, AEs, discounting and consultation lead to only a minor influence on the ICER in contrast to the cabozantinib price and utility values.

Limiting therapy to 4 months led to an ICER of $144,874/LY ($177,314/QALY) regarding the German model and $461,193/LY ($564,461/QALY) for the US model and, assuming progression 1 month after cabozantinib discontinuation, to an ICER of $289,360/LY ($354,153/QALY) for the German model and $897,833/LY ($1,098,933/QALY) for the US model. The ICERs of multiple testing on QoL are presented in Table 5.

**Table 5:** Utility sensitivity analysis

	Utilities				Incremental effectiveness in gained QALYs	ICER in $/QALY	
	Cabozantinib		BSC			German model	US model
	stable	prog	stable	prog			
Base Case [20, 21, 23]	0.760	0.680	0.760	0.680	0.15	375,470	1,189,706
Base Case increased by findings of Abou-Alfa et al. [19]	0.852	0.680	0.760	0.680	0.20	266,479	844,359
Cabozantinib as first-line in advanced RCC [29]	0.817	0.777	0.817	0.777	0.15	362,825	1,149,640
Base case adjusted by AE caused disutilities^a^ [30, 31]	0.728	0.680	0.751	0.680	0.13	427,215	1,353,665
Base case with constantly decreasing utility after progression^b^	0.760	0.680	0.760	0.680	0.14	387,439	1,227,631

*prog* progressive, *BSC* best supportive care, *QALY* quality-adjusted life years, *ICER* incremental cost-effectiveness ratio, *AE* Adverse event. ^a^ The detailed estimations are presented in the Additonal file 1. ^b^ Utilities decreased 0.005 per month for both groups

**Fig. 4:**
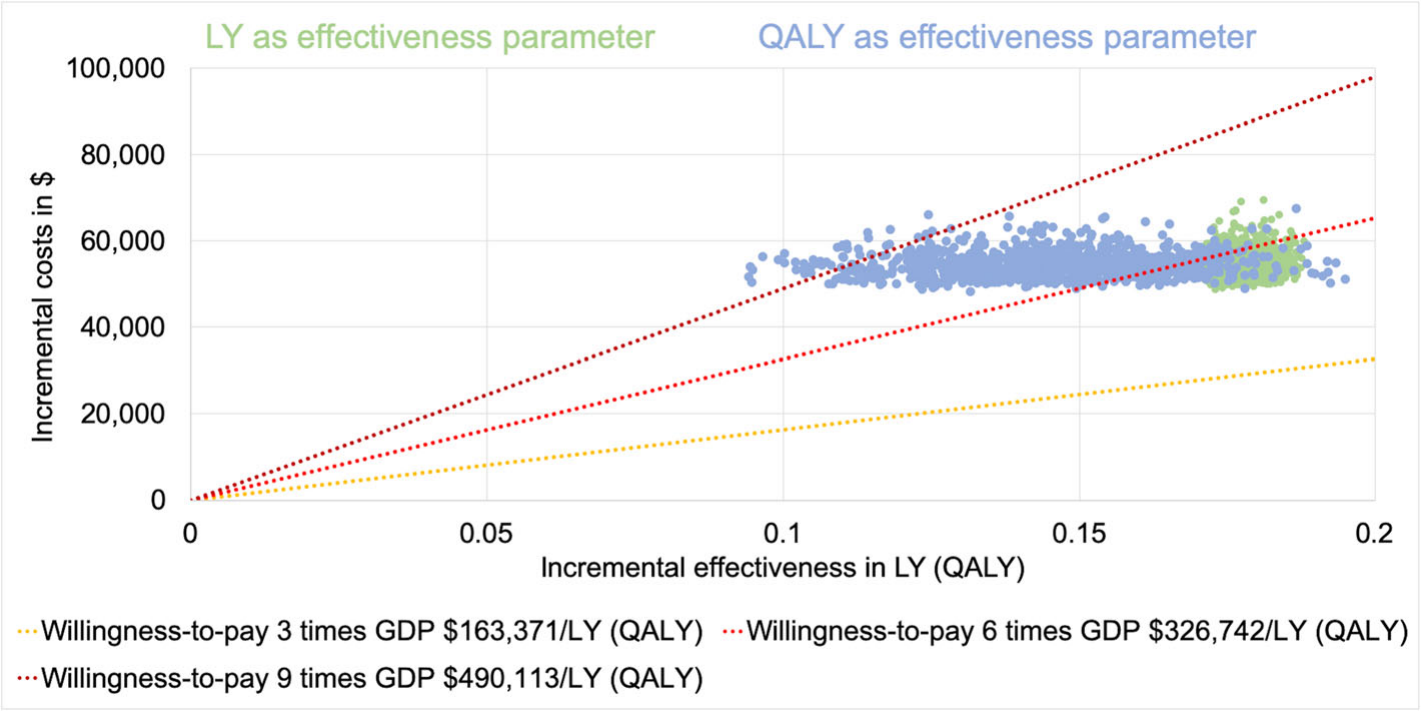
ICER scatter plot German model. GDP = gross domestic product per capita; LY = life year; QALY = quality-adjusted life year. Only the first 1000 iterations were plotted for clarity

**Fig. 5:**
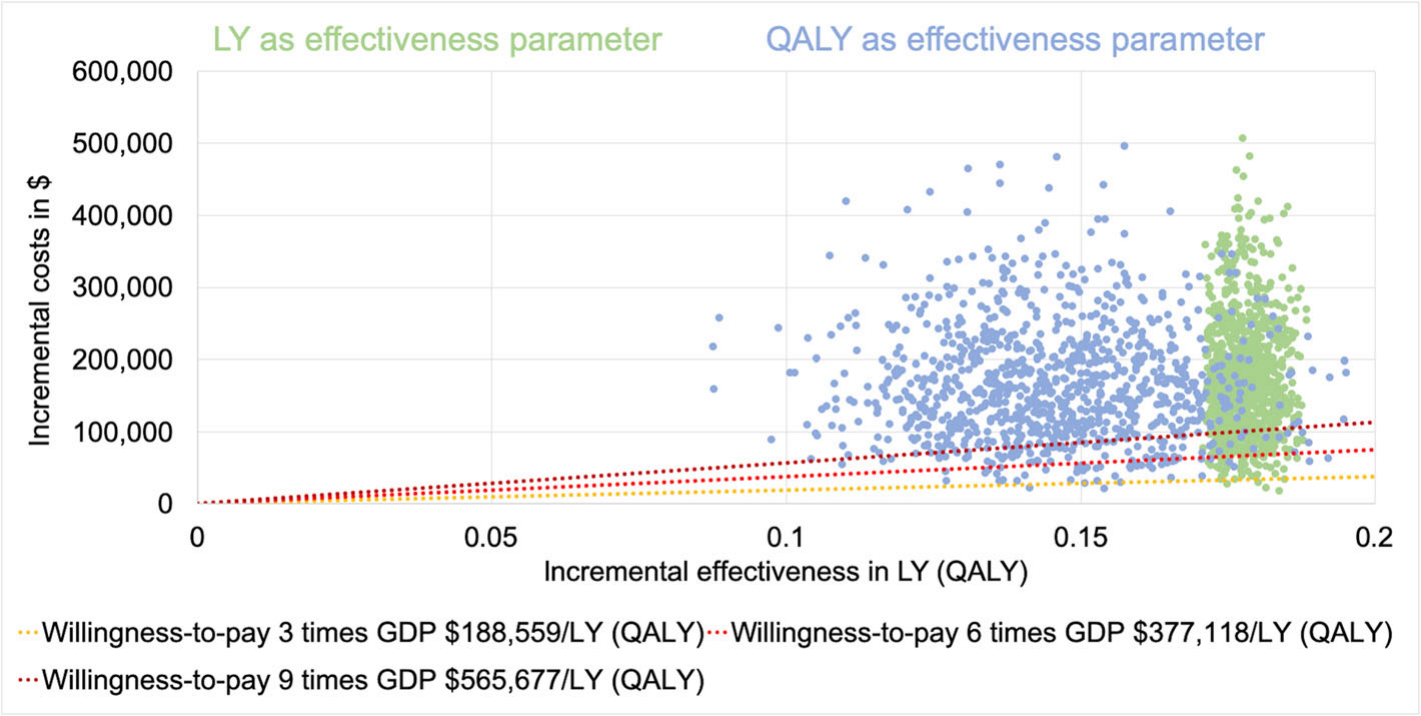
ICER scatter plot US model. GDP = gross domestic product per capita; LY = life year; QALY = quality-adjusted life year. Only the first 1000 iterations were plotted for clarity

The mean results of our probabilistic sensitivity analysis were illustrated through an ICER scatter plot (Figs. 4 and 5). The probabilities of achieving the determined cost-effectiveness thresholds are presented in Table 6.

**Table 6:** Probability of achieving the cost-effectiveness thresholds

cost-effectiveness threshold in GDP per capita/LY (QALY)	Probability of achieving the cost-effectiveness threshold in percent			
	Germany		United States	
	LY	QALY	LY	QALY
3	0	0	0.5	0.2
6	87.3	85.6	5.6	4.3
9	100.0	96.4	19.2	11.7

*GDP* gross domestic product, *LY* life year, *QALY* quality-adjusted life year

## Discussion

Treating HCC after failed sorafenib therapy is effective in our base case with a mean survival gain of 2.2 months versus BSC, supporting the findings of Abou-Alfa et al. and the IQWiG rating of the survival benefit as considerably downgraded to minor owing to the high AE rate [9]. All our scenarios and iterations of our probability analyses showed superior effectiveness and much higher costs of cabozantinib therapy compared with BSC. The base case ICERs were not cost-effective using the 3 times GDP per capita/LY (QALY) thresholds of both models. Cabozantinib drug proportion represented the main cost driver generating over 90% of all costs and showed that combined with QoL utilities, it had the largest impact on ICER in the univariant sensitivity analysis. In addition, our cost-threshold analysis indicates that cabozantinib requires high rebates, reducing its list price to be cost-effective.

The high rates of AEs during placebo therapy underline the massive disease burden of HCC even in populations with Eastern Cooperative Oncology Group score 0 or 1 performance status. In our analysis, AEs induced an increment of $1232 (Germany) and $4955 (US) between the groups but represented, together with other additional costs, less than 10% of the treatment-related costs.

Our analysis has considerable limitations, which we sought to address through our sensitivity analyses. First, a fundamental limitation of our study was the reliance on published data from the CELESTIAL trial. In 2016, the average age of HCC onset was approximately 70 in Germany [3]. Therefore, the real-world effectiveness of cabozantinib might be worse. Further, the lack of suitable QoL data was solved by testing multiple approaches, but all examined combinations were not cost-effective. The relevance for therapy decisions, e.g., between cabozantinib or regorafenib, and the massive impact on cost-effectiveness indicate the need for more standardized QoL reporting in large intervention trials. Ultimately, the practical value for decision makers of our threshold is very unclear, as the GBA has other approaches for assessing cost-effectiveness, and willingness-to-pay remains difficult to measure [32]. Assuming higher cost-effectiveness thresholds led especially in the German model to increased probabilities that cabozantinib is a cost-effective therapy.

IPSEN Pharma refused to submit a cost-effectiveness analysis to the NICE about cabozantinib for HCC because no scenario reached the cost-effectiveness thresholds [33]. In July 2016, the National Health Service (NHS) England established the Cancer Drugs Fund to provide early access to new therapies with unclear effectiveness, but cabozantinib for HCC is not in the current list for January 2020, meaning no reimbursement of cabozantinib [34, 35]. There are two cost-effectiveness analyses of second-line therapy for HCC from the US payer perspective with non-cost-effective ICERs ranging from $469,374/QALY to $1,040,675/QALY [36–38]. These findings and our US pricing scenario match the development of the United States financing the most expensive healthcare system of all countries of the Organisation for Economic Co-operation and Development with expenditures of 16.9% of the gross domestic product (Germany 11.2%) [39]. The high difference ICERs between the US and German scenarios is mostly caused by US drug prices, which are often much higher than German prices (Table 2). Facing various new patented drugs, German lawmakers set maximum prices and statutory rebates via the AMNOG to limit annual drug spending increases [40]. In consequence, the prescription drug price index decreased 15% from 2004 to 2018, estimated after statistics of the AOK Research Institute [41]. In contrast, the US prescription drug price index increased 56% in the same period but decreased in 2019 as the first time since 1974, estimated after statistics of the US Bureau of Labor Statistics [42].

Regorafenib, with its similar survival outcomes, was investigated by two cost-effectiveness analyses, none of which found cost-effective thresholds with ICERs ranging from $201,797 to $277,463/QALY compared with BSC from US payer perspective [43, 44]. The comparability to our US price scenario is limited because these analyses used the much lower drug prices of 2017 and before. As the average cash price of a monthly regorafenib dose in April 2019 was $22,149 according to GoodRx.com (cabozantinib $21,581), current ICERs might be more similar.

Treating advanced medullary thyroid carcinoma with cabozantinib was incorporated in the NHS catalogue after the NICE assessed an ICER versus BSC lower than its cost-effectiveness threshold of £30,000/QALY ($43,541/QALY) [45]. In contrast, the review and economic model of Tappenden et al. found an ICER >£138,000/QALY (>$200,290/QALY) for this setting [46]. Despite health economic analyses regarding cabozantinib as second-line therapy for RCC, its secondary approved indications are divergent. While three cost-effectiveness analyses and the NICE assessment found that cabozantinib dominates versus nivolumab, Deniz et al. investigated that sequences including nivolumab [29, 47–50]. After it was also approved for the first-line treatment of intermediate and high risk RCC, the NICE and Skentzou et al. assessed it as a cost-effective alternative for sunitinib and pazopanib [51, 52].

Introducing expensive new therapies into advanced disease settings as subsequent options might have a large budget impact. Underlining the controversy regarding regorafenib for the third-line treatment of advanced colorectal carcinoma in Germany, Goldstein et al. and Cho et al. reported ICERs of $900,000/QALY and $395,223/QALY versus BSC from US payer perspective [53, 54]. The chimeric antigen receptor T cell therapy for relapsed or refractory pediatric B cell leukemia with its one-time infusion cost of $475,000 was investigated by Lin et al., estimating an ICER of $184,000/QALY versus blinatumomab or chemotherapy from a US payer perspective [55]. The NICE also assessed tisagenlecleucel as not cost-effective but recommended its reimbursement by the Cancer Drugs Fund [56]. Considering all the described findings, our results rank in the upper ICER range of new therapies.

In 2019, there were multiple ongoing phase 3 trials investigating other TKIs or checkpoint inhibitors for HCC [57, 58]. After nivolumab demonstrated effectiveness in a phase 1/2 trial as second-line therapy for HCC, a phase 3 trial for first-line therapy will finish in 2020. Additionally, the impact of the sequential or combined use of TKIs and checkpoint inhibitors will be evaluated, e.g., through a phase 3 trial of cabozantinib and atezolizumab as first-line therapy for HCC [59].

## Conclusion

In conclusion, cabozantinib is an effective but not cost-effective second-line therapy for HCC, as evaluated in two different healthcare systems. Our primary cost-effectiveness threshold was not achieved in our base cases or almost all our scenarios. The German model had a higher probability to reach the cost-effectiveness thresholds than the US model caused by the much higher drug prices. The main cost driver is the cabozantinib drug price, and the highest uncertainty arises from QoL inputs.

## Supplementary information

**Additional file 1 Supporting data file.** This excel file contains cited supporting information and supplementary material.

## Data Availability

All data generated or analyzed during this study are included in this published article and its supplementary information files.

## Abbreviations

HCC: hepatocellular carcinoma
OS: overall survival
EMA: European Medicines Agency
GBA: Federal Joint Committee
SHI: statutory health insurances
IQWiG: German Institute for Quality and Efficiency in Health Care
TKI: tyrosine kinase inhibitor
VEGFR: vascular endothelial growth factor receptor
MET: hepatocyte growth factor receptor
RCC: renal cell carcinomas
PFS: progression-free survival
BSC: best supportive care
ICER: incremental cost-effectiveness ratio
LY: life year
QALY: quality-adjusted life year
GDP: gross domestic product
QoL: quality of life
AE: adverse event
NICE: British National Institute for Health and Care Excellence
DRG: diagnosis related groups
AMNOG: Pharmaceuticals Market Reorganization Act
NHS: National Health Service
